# Hospital and Institutional News

**Published:** 1915-02-20

**Authors:** 


### HOSPITAL AND INSTITUTIONAL NEWS.

### THE EXCHANGE OF DOCTOR AND INVALID PRISONERS.

On Monday last, in accordance with an agreement for the release by this country and by the enemy of all incapacitated military prisoners, the first batch of Germans, whose serious wounds or amputated limbs incapacitated them for further active service, left London for Germany. Ninety-four in number and drawn from hospitals all over the country, the prisoners, according to a War Office selection, were in charge of five medical men and a detachment of thirty City of London Red Cross workers under Lord Onslow. From Victoria a Red Cross train carried them to Folkestone, where they were assisted by two supporting attendants, or lowered on stretchers to the vessel, which was bound for Flushing. The party was to be exchanged, as Mr. Neil Primrose pointed out in the House of Commons on Monday in reply to questions, for a similar number of British wounded prisoners, according to official information received by the Government on February 13. The notice given was, he added, too short to allow of all the thorough arrangements for their comfort which the Government would have liked to make, and, as it turned out, our wounded were detained one day for some reason or other. Their arrival should give us unimpeachable evidence of the conditions which have prevailed in their places of detention, although, as seriously wounded, they are likely to have been, we hope, exceptionally well cared for. Mr. Neil Primrose also stated that an agreement for the mutual release of doctors had also been concluded. We trust, therefore, that a policy which has everything to recommend it, and nothing objectionable from a belligerent's point of view, may now be carried out with reasonable promptitude and regularity by both sides. The Government's attempt to gain German agreement for the mutual release of interned invalid civilians has so far failed.

### COMMISSION FOR A HOSPITAL SECRETARY.

The appointment was announced last Saturday to a commission as lieutenant of Mr. Louis C. McCausland, the secretary of the Kensington and Fulham General Hospital. He has joined the 22nd (Service) Battalion (Kensington) Royal Fusiliers, City of London Regiment, which is in training at Horsham. The Kensington institution, since the war began, has sent several members of its staff abroad. The matron of the hospital is in charge of a nursing institution at Boulogne, and two surgeons, the skin specialists and several medicos are at the Front.

### WOUNDED SOLDIERS AND THE EPSOM SPRING MEETING.

Soon after the outbreak of war, in the first rush of enthusiasm when every type of public and private building was being offered for hospital purposes, the Epsom Grand Stand, belonging to the Grand Stand Association, was conditionally accepted for this purpose. It has been used for several months as a hospital for wounded soldiers, and, according to Mr. Tennant in the House of Commons, arrangements were made by the Epsom Hospital Committee to vacate the building in time for the spring meeting. This involves, of course, the removal of the wounded, who, it is understood, have gained much from the situation of the hospital and the satisfactory character of the work. Some forty wounded soldiers are now in the hospital, and questions have been asked as to whether their removal is not attended with risk. In view of the agreement referred to above, the responsible hospital committee has presumably arranged its cases so that the building can be vacated without danger; but that presumption does not alter the fact that to take a building for a hospital for wounded, especially a grand-stand annex, with the proviso to remove the men on the eve of the race meetings, has an atmosphere not likely to be approved of just now. We think, then, the original arrangement open to objection, and trust that the responsible authorities, while using, if possible, the neighbourhood's advantages, will in future disapprove of all agreements made for short terms in buildings periodically booked for public amusement. A quite unnecessary feeling of friction and occasion for comment has been created which could have been avoided had not the committee apparently supposed that, after all, the men would be allowed to stay for the spring meeting at Epsom, a course about as open to objection as their removal.

### PERSONAL SERVICE FOR THE WOUNDED.

The response to the proposal to form a Voluntary Hospitals Brigade to render personal service, by undertaking the collection of contributions from the small givers for the benefit of the voluntary hospitals, has been remarkable and cheering. Everyone will agree that it is desirable not to multiply organisations for raising funds in connection with the present war. It is a pleasure, therefore, to announce that the work already initiated will be carried on through the League of Mercy, which is collecting funds to assist in defraying the cost of treatment of the sick and wounded in our voluntary hospitals. The principle of the League is to distribute through the King's Fund all the money raised through its Metropolitan branches, and to devote an increasing portion of the collections made in the Home Counties to the local voluntary hospitals. Offers of personal service have been received from residents throughout the United Kingdom. The Grand President of the League of Mercy has created a new division of the League for the United Kingdom outside the existing districts, which should meet this influx of new workers, so that everyone willing to render personal service for the sick and wounded can at once become members of the League. It is hoped that the large number of workers who have already tendered their services may be increased by a hundredfold within the next month. Many who have found continuous knitting an arduous task may seek relief by becoming members of the League of Mercy, and undertaking each to raise at least £1 a year in small subscriptions of 10s. and under. If the interest already manifested in this scheme is maintained, at least 100,000 new workers should speedily be enrolled. Everyone, therefore, willing to render personal service, to however limited an extent, by becoming a member of the League of Mercy, should write to the Editor at once.

### THE ABOLITION OF OUTSIDE SICK VISITING.

Matters of genuine interest were transacted at the recent annual meeting of the Peterborough Infirmary and Dispensary, which will probably make it stand out as an important date in the history of the institution. For instance, a new source of income was reported in an item of £15 odd from approved societies for insured persons who have received treatment, an item which it has been the fortune of extremely few hospitals to record. Another interesting point was the confirmation by the governors of the action of the quarterly committee in October that all Belgian refugees who produced a letter from a responsible person as to their *bona fides* should receive free treatment at the infirmary. It does not appear if any or how many Belgians took advantage of this action, but it is not easy to recall instances at the moment in which a similar decision has been made. Owing to the shortage of medical men, it had been found impossible to secure a man as house surgeon even at twice the former salary, but eventually a woman house surgeon, Miss Jane Filshill, was found. A special difficulty was complained of owing to the fact that they could not get men to accept such an appointment so long as out-visiting was attached to it. Indeed, Miss Filshill had said that she must give up the work if out-visiting was still to be continued.

### SURGICAL INSTRUCTION SIXTY YEARS AGO.

On this head Dr. T. J. Walker, consulting surgeon, had some interesting recollections. After pointing out that the hospital was originally the sick-house attached to a dispensary, he stated that the visiting work had now multiplied, he might say, a thousandfold. He recalled that sixty years ago, when he was first articled to his father, part of his instruction was to go round visiting the cases with the house surgeon. Now that visiting had almost disappeared, while the work in hospital had enormously increased. It was a serious step to cut off one part of the infirmary, but out-visiting took the house surgeon away from his or her proper duties. If it were not abolished, either the staff must be increased or the hospital rebuilt. The poor would not be hurt by its abolition, for visiting the sick had been taken over by public bodies and provided for by the Insurance Act. Operations had gone up from one to eight hundred a year, and yet there was only one house surgeon as in 1879. Properly discharged, it was a most strenuous appointment. Needless to say, its abolition was agreed to, and an old order of things has passed away. But, as it is passed, we may record one good thing about this despised out-visiting, that from the keen young practitioner's point of view, tiresome though it was, it gave him clinical experience very valuable afterwards in general practice, since it is the small, or chronic, ailments, rather than the "interesting cases," which take up the bulk of the general practitioner's time.

### THE POST OF DISPENSER, SECRETARY, AND REGISTRAR.

Another important change in the laws of Peterborough Infirmary was decided on at the same meeting of governors. Not only was the dual work of the house surgeon, outside sick visiting as well as the hospital duties, done away by the abolition of the former, but the dual posts of secretary and dispenser were likewise separated, though each was retained. A dispenser is to be appointed. In the past the secretary had been not only dispenser but also registrar, with the result that his health had broken down under the strain. Dr. Walker, consulting surgeon, spoke on this subject and said that he had watched the secretary's work for thirty-five years, and that one man could no longer carry out this work in addition to other duties. Mr. A. C. Taylor, the secretary, is therefore a link between the old system and the new, and his term will always stand out as that which witnessed the abolition of the threefold office, and the limitation of the chief lay administrator's office to its own strictly defined but largely increased sphere of work. We believe that he will lay aside this ancient triple diadem without regret.

### THE CARDIFF SOCIETY FOR AIDING RELATIVES.

The Society at Cardiff which was inaugurated to enable relatives of sailors and soldiers to visit them has established a successful procedure. The effect of the reunions upon the men's spirits is wonderful. The secretary of this organisation is the ex-Lady Mayoress, Mrs. Robinson, who co-operates with the commanding officer, Colonel Hepburn, and the adjutant and registrar, Major Maclean, of the 3rd Western General Hospital. Whenever it is thought desirable to send for a relative Mrs. Robinson writes a personal letter to the woman—mother, wife, or whoever she is—acquainting her of the patient's wish, and telling her that her fare will be paid and hospitality found her at Cardiff. She is met on her arrival by one of the Women's Emergency Corps, wearing a distinctive badge on her left arm, and the visitor is taken to the hospital and afterwards to her lodging. Mrs. Robinson has written to the mayor of almost every military town in the kingdom telling them what is done in the Glamorgan capital, with the hope that the example will be emulated.

### THE COTTAGE HOSPITAL SECRETARY.

Few hospital officials are really more hard-working than those honorary secretaries of the smaller institutions who are secretaries in fact as well as in name, and have little or no assistance in the work for which they make themselves voluntarily responsible. There is always a period in the growth of the cottage hospital when the question of a paid secretary looms ahead, but which is shelved so long, and it is often surprisingly long, as the honorary secretary loyally struggles with increasing demands on his time and energy. In this connection we may note the tribute recently paid to the work of Mr. H. A. Perkins, honorary secretary to the Queen Victoria Cottage Hospital, East Grinstead. The chairman and the representative of the working men's hospital fund alike commended his work. His reply bore out our contention, for he said that his heart was in it, and we believe this to be most characteristically the case in the best administered of the small hospitals where voluntary work of busy men is the mainstay of the institution.

### BRIDGE FOR CHARITY: THE SUBSCRIBERS' POINT OF VIEW.

With reference to a paragraph under the above heading, which recorded last week a bridge tournament organised on behalf of the Samaritan Free Hospital for Women, a correspondent writes: "Two years ago a titled and fashionable lady, who owns a big house in Eaton Square, gave a bridge party and advertised that the proceeds would be given to a certain well-known hospital. In due course the hospital received about £38, being the proceeds of the bridge party. At the end of the year the hospital published its usual annual report, and on one of the pages was a two-line reference to the bridge party and its proceeds. Within six months, at least a score of letters were received in which bridge for charity was severely condemned. The majority of the writers of the letters were elderly lady subscribers to the hospital, who said that if 'gambling' money was to be used as a means of maintaining the hospital they would discontinue their help. Whether their attitude was narrow-minded it does not matter much, but the fact remains that hospitals cannot be too careful in announcing the sources of their income. Doubtless some of those who object to bridge for charity will be influenced by their prejudice when they come to make their wills. In any case, it is important that those who send appeals broadcast should walk very warily and should prepare their reports with the utmost care and thought." We would go further than our correspondent and ask: If there are certain sources of income which it is unwise to announce, is not the inference in the majority of cases that the source itself should be dispensed with?

### A MISSING HOSPITAL OFFICER FOUND.

A body, believed to be that of Mr. Oliver Hiscutt, the missing assistant curator of the museum at the London Hospital Medical School, was recovered from the Thames, near Barnes, last Saturday afternoon by the river police. On being removed to the mortuary, certain articles found in the pockets were identified by a representative of the London Hospital and by Mr. Hiscutt's brothers-in-law. Among them were two pocket-handkerchiefs, which were marked in the corner with the name "Hiscutt," and also a bunch of keys, which were identified afterwards as being those belonging to the London Hospital Medical School museum. Some loose change and an evening newspaper dated January 14 were also found. The body, which is understood to have borne no marks of violence, had, apparently, been in the water between three and four weeks, and was unrecognisable.

### DOCTORS' FEES FOR EXAMINING RECRUITS.

Thirty-six medical practitioners in Sunderland are reported to have contributed to the Mayor of Sunderland's Fund, associated with the Prince of Wales's National Relief Fund, the fees which have been received by them for their work in connection with the examination of recruits. These fees amounted to over £230, and the Prince's Fund has now reached rather more than four and a half million. While appreciating the generosity of the medical men concerned and recognising their right to devote their charitable gifts in what direction seems best to their knowledge, we may venture to wonder that they should have preferred to augment a fund for the general relief of distress to aiding the really splendid work for the nation which the voluntary hospitals, where they themselves were trained, are now doing with virtually no reward. Is there any reason why medical men who feel that the fees which they receive for examining recruits should be given to charity, should ignore the claims of voluntary hospitals at the present time? That the above view is based on common-sense as well as on the old sentiment that charity should begin at home, may be shown by the following facts. The National Relief Fund has reached an enormous total; the funds already subscribed are very largely unspent—Mr. Herbert Samuel last week put the unexpended proportion at four-fifths; while on the other hand the hospitals which are receiving wounded have no unexpended special funds at their disposal, and all indications show that the work in this direction will still further increase with the prosecution of the spring campaign. Under these circumstances we think that the doctors' generosity was more conspicuous than satisfactory, and venture to point out to any other medical men equally benevolent a nearer and still more pressing claim to their charity.

### HONOUR FOR A MEDICAL SUPERINTENDENT.

Dr. Spencer Mort, M.D., F.R.C.S.(Ed.), medical superintendent of Edmonton Infirmary, London, has been invited to take charge of a large hospital in France. The hospital is under the control of the Red Cross Society of the French Government. At a recent meeting of the Edmonton Board of Guardians it was decided to give Dr. Mort three months' leave of absence to enable him to undertake this work. The Guardians consider the selection of Dr. Mort is a distinct compliment not only to him but to the Board. Dr. Mort had a distinguished career as a student, obtaining the "Brunton" Prize, "Hunter" Medal for Surgery, and "Cullen" Medal for Medicine, and during the few years he has had control of the infirmary at Edmonton has done much useful work. In the early stages of the war, this infirmary proved very helpful in treating sick Belgian refugees who were transferred to Edmonton from the Alexandra Palace.

### SHOULD A VERANDAH COUNT AS FLOOR SPACE?

The London County Council recently built at Bexley Mental Hospital a villa to accommodate 30 male phthisical and dysenteric patients and attached to it is a verandah, 13½ feet wide and 113 feet long. The Board of Control will not as a rule permit verandah accommodation to be considered part of the regulation dormitory or day room space which has to be allowed for each patient, and the area of the verandah was, therefore, not computed in the accommodation when the plans were approved by the Home Secretary. The accommodation provided in this villa is not, however, partitioned into dormitory or day room space, but the whole of it is constantly in use and serves both purposes. The verandah forms an integral part of the villa, and the inclusion of its area as part of the recognised floor space would enable accommodation to be provided for 12 more patients, raising the total accommodation of the villa from 30 to 42 beds. The Board of Control have now intimated that they will raise no objection to the space being regarded as part of the ward and to the accommodation being thus far increased beyond what was originally intended, provided that only patients suffering from diseases for which the building was set apart are located there. It will be recalled how the verandahs at Queen Mary's Hospital for Children, Carshalton, were found doubly useful as securing open-air treatment, while also extending the accommodation of each cottage block.

### THE LIST OF LOWEST PRICES.

The Council of the King's Fund are seriously considering the advisability of discontinuing to issue the "List of Lowest Prices," which has appeared annually in March for some years past. A circular-letter has been issued by the Fund to participating hospitals asking for opinions and advice as to whether the list should be discontinued altogether, or, alternatively, whether it should be omitted this year when the general upheaval of prices has rendered comparisons of little value. The list has usually contained the prices paid by more than 100 hospitals for about 50 articles, and it has naturally demanded much time and trouble in its preparation not only on the part of the Fund, but also on the part of hospitals supplying the information. Its purpose was, in the Fund's own words, "to assist hospitals in comparing their own prices with those paid at other hospitals, and thus to facilitate their inquiries into one of the factors affecting the cost of working, as shown in the Statistical Tables." But the Fund confesses to find that the enterprise has met with scanty success. In the forms sent out to be filled up last year, the scope of the inquiries was considerably extended, the important points of quality, length of contract, and date of purchase being included for the first time. This extra information was not, however, published in the digest circulated amongst the hospitals, but the original forms were made available for inspection at the offices of the Fund. They were not consulted in a single instance. It is obvious, therefore, either that the hospitals do not appreciate the value of the list, or that it fails in practical utility to them. Which is the case?

### BUILDING SCHEMES AND UNEMPLOYMENT.

Lord Provost Taggart presided last week over the annual meeting of the corporation of the Royal Infirmary and Mental Hospital of Aberdeen, when Mr. Alexander Ledingham had some interesting remarks to make about the institution. The Mental Hospital happened to be one of those which, in December 1913, contracted for the greater part of its supplies for the whole year, and, consequently, in some degree, escaped the higher prices which followed on the outbreak of war. The great building scheme, begun twenty-two years ago, continues gradually to be carried out; the new kitchens were completed six months ago, but the recreation rooms and dining halls have still to be undertaken, while a block of day rooms and dormitories some thirty-five years old has to be refitted. The question has here again risen as to how far building operations should be continued under present circumstances, but opinion seems to favour a gradual continuance of the work. The debt, it is true, amounts to over £70,000, but it is hoped to pay this off in the next thirty years, by which time the present reconstructed buildings will probably be due for overhauling once more. From the point of view of a public body the possibility of considerable unemployment after the war has also to be thought of, and this will certainly arise if the first advertised policy of discharging men with all convenient speed as soon as the war is over is to be carried out. Indeed, it may be prophesied that any attempts in this direction would prove so disastrous that some better way would have to be found than spending the surplus millions of the relief funds on the victims of such a policy. Still, the future of institutional building schemes cannot be entirely decided by considerations which lie within the direct province of the Government; and, so far as possible, it is desirable to carry out the plans decided on.

### THE SPECIAL HOSPITAL FOR WOUNDED MEN.

In previous issues of The Hospital we have recorded not only the opening of the Special Hospital for Officers suffering from shock, but also the decision of the War Office to open a similar institution for similar cases among the men. Now known as the Lancashire Military Hospital, information has been given in the House of Commons on the subject of the administration. In the hospital, which is, of course, an exclusively military institution, the reception, treatment, and discharge of soldiers admitted are under the sole control of the War Office. While the soldiers, Mr. Tennant pointed out, are not under the jurisdiction of any mental authority, the premises of the hospital are the property of the Board of Control. Members of the Board periodically visit the institution to supervise such matters as supplies, heating, repairs, and maintenance, but they in no way concern themselves with the treatment of the patients. This information may serve to dispose of any misunderstandings which have been prevalent in the lay mind about the institution, but it does not add much to the knowledge of institutional officers, who realise that the class of case, as a whole, is one which requires a special institution, and in the main is quite distinct from those with which the Board of Control is concerned.

### THE WAR AND THE CORONERS.

The effect of the war on the work of Coroners has been to make them much busier than before, remarked Dr. Waldo at a recent inquest. It has also modified the manner in which they are to interpret the first section of the Coroners Act. According to this section all violent deaths must be inquired into, but since the beginning of the war Dr. Waldo remarked that he had had the deaths of six wounded soldiers reported to him. After very full inquiries he had decided that it was unnecessary to hold inquests. In fact, Mr. McKenna had stated that Coroners should not hold inquests in such cases, and the Lord Chancellor had agreed with him, notwithstanding the Coroners Act. The action of the Lord Chancellor, Dr. Waldo remarked, had relieved him of responsibility, but notwithstanding, as an outcome of the war, Coroners are much more busy than usual.

### THE MAINTENANCE OF MILITARY PATIENTS.

We hope to publish next week a technical illustrated description of the new ward for wounded soldiers which has been put up at the Norfolk and Norwich Hospital. Opened on Thursday last week, the new ward with its sixty beds is interesting as one of those emergency or temporary buildings which in time of peace have often been recommended for hospital purposes, and on which we have had to rely so extensively during the present European war. The expenses of maintenance, which, however, call for immediate notice, are being met in an ingenious way. An appeal on this head was issued and contained the suggestion that individuals or local firms should maintain them by means of a monthly subscription of £5. Already of the sixty beds no fewer than forty have cards indicating the name of the person or firm which has become responsible for it under the maintenance scheme. The idea, in short, has proved a great success, and may be commended to all hospital areas where it has not already been tried and to which a constant stimulus to work for is desirable.

### MEDICAL OFFICER OF HEALTH AND MILITARY DUTY.

Dr. G. Q. Lennane, F.R.C.S., medical officer of health for Battersea, has been gazetted a lieutenant in the 2nd London Sanitary Company R.A.M.C., Territorial Force. For the present he is able to attend to his civil duties, but later on it is anticipated that his full services will be required, and he will probably have to go to the Front. The Borough Council have decided to pay Dr. Lennane the difference between his civil pay and his Service pay and allowances, and to engage the services of a *locum tenens* during his absence. The matter caused considerable debate when it was before the Council. It was felt by a section of the Councillors that Dr. Lennane's services were of more value to the borough, and that other medical men could be found who could perform his duty with the Sanitary Company. Whilst he was away it was realised that the services of another medical man to act in the interests of the borough would have to be obtained, and it was this extra expenditure that would have to be met by the Council that was most objected to. There is little doubt that the people of Battersea would have given Dr. Lennane permission to go without the slightest demur, providing they knew that his services were essential to the health of the troops, even though the inhabitants of the borough had to suffer through his absence. Dr. Lennane has been eleven years medical officer of health at Battersea, and has done much to improve the conditions under which the population of 170,000 people live.

### A HOSPITAL FOR PERMANENTLY DISABLED BELGIANS.

Princess Clementine of Belgium paid a visit to Cheltenham last week in order to open Rothesay House as a hospital for Belgians who have become permanently disabled. The actual beginning of work will depend upon the completion of the equipment, when the patients will begin to arrive. On the occasion of the visit the Belgians from other hospitals in Cheltenham assembled at the New Court Red Cross Hospital, where Lord and Lady Bathurst received the Princess. Efforts are being made to raise the necessary funds as soon as possible, and the institution is probably the latest example of that specialised work which has already produced such a crop of special institutions since the war began. The hospital claims to be the first of its kind in England, and, if it is properly supported and maintained, should have the value of collecting the men who have been crippled in their country's service at one centre—that is, under circumstances which should guard against the too familiar scandals of neglect in the past.

### IRELAND'S PROVISION FOR THE WOUNDED.

On the occasion of the opening of the County Wicklow ward for wounded soldiers last week at Sir Patrick Dun's Hospital, Dublin, Sir Charles Ball made some interesting observations on Ireland's hospital accommodation for the wounded. He had, he said, with his colleague, Sir Thomas Myles, spent two months in inspecting the hospital provision available not only in Dublin, but in Belfast and Cork and elsewhere. Taking the military hospitals, there were a great many, but only three or four fully equipped for really serious cases.

Those institutions, in fact, were established on the basis of an Army as it existed before the war; but the number of soldiers now in Ireland was close on double that during peace, and so these hospitals were kept occupied to the uttermost in relieving ordinary accidents and illness. If there were a large influx of wounded from the Continent the military hospitals could not accommodate them. The Director-General had told him that they had on a list over a thousand hospitals which had offered to take in the wounded. All the clinical hospitals in Dublin, Cork, and Belfast had thrown open their wards, in fact in some cases he thought that local claims had suffered too much. Next came the new institutions; costly to equip, not as convenient as the clinical hospitals, but necessary. Special hospitals had also provided their quota of beds for suitable cases. For the less serious cases they had a number of small hospitals and county infirmaries. Then came the convalescent homes, or, as they were now called, transition hospitals, owing to some red-tape about the furlough of the men. As he understood matters, there were some forty wounded soldiers of the last batch being treated in Dublin, and six hundred beds were available if the wounded were to arrive to-morrow. The provision in Ireland, he thought, would now be sufficient.

### USEFUL HINTS FOR DISPENSERS.

The discolouration of mixtures containing sodium salicylate and sodium bicarbonate has been a considerable source of trouble to dispensers. It has been attributed to the use of a stock solution of sodium bicarbonate in which part of the bicarbonate had been converted into carbonate, the action of the carbonate on the salicylate being more marked than that of the bicarbonate. The matter has been investigated by Professor H. G. Greenish and Mr. A. E. Beesley in the Pharmaceutical Society's Research Laboratory, and the result of the investigations communicated at a meeting of the Society on February 9. The conclusion arrived at is that the discolouration appears to be due to the oxygen of the air acting on the salicylate in the presence of sodium sesquicarbonate. The addition of about a grain of sodium sulphite or bisulphite to an ordinary eight-ounce mixture produces a marked preservative effect, and it is difficult to see what objection could be raised to such an addition. It is satisfactory to know what the cause of this difficulty really is, and dispensers will no doubt be quick to adopt the means now suggested of avoiding it. The infinitesimal quantity of sodium sulphite could not possibly have any influence on the medicinal properties of the mixture, and it is hardly probable that prescribers would raise any objection to the employment of such a simple expedient for producing an elegant preparation.

### ASPIRIN SUBSTITUTES.

In another paper read at the same meeting, Professor H. L. Smith gave the results of the examination of some samples of acetylsalicylic acid which has been conducted in the Society's Research Laboratory. It appears that doubt has been expressed by some medical men and others as to the identity of the various makes of acetylsalicylic acid, and especially as to their identity with aspirin. Professor Smith finds that this commonly prescriptive acid—which before the war had been procured almost exclusively from Germany—may now be obtained without difficulty, chemically pure, from more than one source; but, as with other chemicals, indifferent and impure makes are on the market, and the same discrimination should be used in buying it as with other drugs.

### CONDITION OF THE MEN AT THE CRYSTAL PALACE.

The Secretary of the Admiralty has issued the following official denial of the very persistent rumours that "spotted fever" is rife among the men of the Naval Division quartered at the Crystal Palace, which was abruptly closed to the public last week: "Rumours in circulation that the general health of the Naval Division in training at the Crystal Palace is unsatisfactory are quite inaccurate. Cases of serious illness of every kind are bound to occur in any large body of men like that of the Naval Division, but on the whole the health of the men has been and is excellent, and there is no reason to anticipate any deterioration in this respect. Out of 8,000 men the average sick list is under 150, or less than 2 per cent." This reassuring statement would have been still further enforced if the latest figures had been given; and it is much to be hoped that they will be forthcoming.

### THIS WEEK'S DRUG MARKET.

The general tendency of the prices of drugs continues to be in an upward direction, and this is especially marked in the case of synthetic drugs. For instance, phenacetin and phenazone are again dearer, and the increased quotations for salicylates are well maintained, with a strong probability of a further increase. Potassium chlorate, potassium permanganate, and potassium carbonate are all dearer, mainly as a result of the announcement that Germany has prohibited the export of potash. Alum and Epsom salts are tending upwards in price. Potassium bromide and other bromides are offered more freely, and values have a slightly downward tendency. A further advance in the price of Epsom salts is almost certain. Cod-liver oil is an uncertain position, and while there is little difference in the quotations since last week, there seems to be a slightly easier feeling in the market, as a result of reports from Norway that the yield of oil may possibly be very good; but it is impossible to forecast the future of this market as there are so many uncertain factors to be taken into account. Quinine is still unchanged, but an advance in price is still expected. Cocaine seems likely to advance in price. Senna leaves are dearer, as also is saffron. Ipecacuanha has a downward tendency in price. Business during the week has been somewhat quieter, but still quite active.

